# Civil Disobedience in Times of Pandemic: Clarifying Rights and Duties

**DOI:** 10.1007/s11572-021-09592-7

**Published:** 2021-07-28

**Authors:** Yoann Della Croce, Ophelia Nicole-Berva

**Affiliations:** 1 Department of Political Science and International Relations, University of Geneva, 40 Boulevard du Pont d’Arve, 1205 Geneva, Switzerland; 2 Department of Political Science and Social Sciences, European University Institute, 9 Via dei Roccettini, 50014 Domenico di Fiesole, Italy

**Keywords:** Civil disobedience, Pandemic, Conctractualism, Care, Rights

## Abstract

This paper seeks to investigate and assess a particular form of relationship between the State and its citizens in the context of the COVID-19 pandemic, namely that of obedience to the law and its related right of protest through civil disobedience. We do so by conducting an analysis and normative evaluation of two cases of disobedience to the law: (1) healthcare professionals refusing to attend work as a protest against unsafe working conditions, and (2) citizens who use public demonstration and deliberately ignore measures of social distancing as a way of protesting against lockdown. While different in many aspects, both are substantially similar with respect to one element: their respective protesters both rely on unlawful actions in order to bring change to a policy they consider unjust. We question the extent to which healthcare professionals may participate in civil disobedience with respect to the duty of care intrinsic to the medical profession, and the extent to which opponents of lockdown and confinement measures may reasonably engage in protests without endangering the lives and basic rights of non-dissenting citizens. Drawing on a contractualist normative framework, our analysis leads us to conclude that while both cases qualify as civil disobedience in the descriptive sense, only the case of healthcare professionals qualifies as morally justified civil disobedience.

## Introduction

So this is a great time, gentlemen and ladies, for civil disobedience. We need to be the Rosa Parks, here, and protest against these government injustices*.*^1^

The current COVID-19 pandemic has raised some serious ethical conundrums regarding, among others, what would be a just allocation of scarce medical resources such as ventilators (Emanuel et al., 2020; Mannelli, 2020; White & Lo, 2020), what principles should be guiding triage policies (Schuklenk, 2020a; Truog et al., 2020), and to what extent may individual privacy be infringed upon for effective containment and tracing of the disease (Parker et al., 2020). There are and undoubtedly will be many more ethical questions to be discussed and hopefully corresponding answers to be found, even regarding the very way in which we should frame these (Celermajer & Nassar, 2020). What we wish to examine in this paper goes beyond ethics in clinical settings and extends to the abstract question of what political participation and protest rights citizens possess during a pandemic and what subsequent measures of containment may legitimately be undertaken by governments.

As one can infer from this opening quote, the pandemic crisis has had a dividing effect in the relationship between citizens and their government. Some citizens apply their State’s measures to perfection and even regret that, in their opinion, they were imposed too late, whereas others like Stephen Moore,^2^ the author of the above sentence, believe that the State is overstepping its power boundaries in restricting individual freedoms in the name of collective health. Demonstrations against confinement measures, albeit modest, began in the United States before spreading to Europe, notably in the United Kingdom (Duncan, 3 May 2020), Germany (Zeit Online, 9 May 2020), and Switzerland (Zünd, 12 May 2020). These protesters, tired of staying at home, isolated, and in a hurry to return to work, are not the only ones to have expressed their dissatisfaction during the current crisis. Nurses and other healthcare professionals have also warned about their unsafe working conditions and lack of protective gear, threatening to refuse to go to work should these conditions not be decent enough to guarantee their patients’ safety as well as their own. Such a situation unfolded exactly in this manner in California, leading to the suspension of the ten protesting nurses (The Guardian, 17 April 2020).

This paper seeks to investigate and assess a particular form of relationship between the State and its citizens in the context of the COVID-19 pandemic, namely that of obedience to the law and its related right of protest through civil disobedience. We do so by conducting an analysis and normative evaluation of the two aforementioned cases of non-compliance with the law. While these cases are indeed individually complex and each of them possesses particular features, they are substantially similar with respect to one element: their respective protesters rely on unlawful actions in order to bring about a change in policy because they consider current policies unjust. As such, we question (1) the extent to which healthcare professionals may participate in civil disobedience with respect to the duty of care intrinsic to the medical profession, and (2) the extent to which opponents of lockdown and confinement measures may reasonably engage in acts of protestation without endangering the lives and basic rights of other, non-dissenting citizens. We proceed by assessing both cases through the prism of civil disobedience in order to know whether or not they effectively possess the features of an act of civil disobedience in the descriptive sense. After having done so, we evaluate whether or not they constitute acts of morally justified civil disobedience. In order to effectively provide a thorough analysis of these two cases, we start by introducing a formalization of the duty not to infect others that draws inspiration from contractualist moral theory. Doing so enables us to derive from the idea of fundamental principles shared by all individuals what we refer to as the *containment principle*, which broadly asserts the health-related rights and duties individuals hold towards each other in times of pandemic. The duty not to infect consists, broadly speaking, in preventing the spread of the virus from one person to another when reasonably possible. In short, we argue that the duty not to infect is a composite of the positive duty of easy rescue and of the normative injunctions that originate from the containment principle. We then introduce the concept of civil disobedience and present its two main features, namely those of *conscientiousness* and *communication*. We then propose a reconstruction of the concept by defining what is meant by *morally justified* civil disobedience, with the help of our contractualist account regarding the ethics of contagion and containment. We then apply our theoretical apparatus to the cases of essential workers, specifically supermarket clerks, who refuse to attend work which helps us in establishing a starting point for the case of healthcare professionals—and confinement opponents who protest through public demonstration, concluding that while both of them qualify as civil disobedience in the descriptive sense, only the case of healthcare professionals qualifies as morally justified civil disobedience.

## Contractualism, Health Rights, and Civil Disobedience

### Contractualism and the Containment Principle

Lockdown and confinement measures are not merely justified in virtue of pragmatic and factual elements or of an appeal to a vague idea of public safety. Since these measures necessarily imply a substantive restriction on fundamental rights, such as the freedom of movement, association, or assembly, they ought to be justified according to another corresponding fundamental moral right; or in other words, it must be shown that there are sufficiently strong moral grounds that justify confinement measures in order to justify a restriction on freedom of movement. In order to flesh out this justification we turn to contractualist moral theory.

Contractualist theory is ultimately grounded in the equal moral status of all persons. As such, the theory helps us frame the moral basis of confinement measures as a set of relations between one’s rights and duties and the rights and duties of others, where these rights and duties have been clearly defined under conditions of mutual agreement. Contractualism states that an act is to be considered wrong if it may be reasonably forbidden according to principles that have been reached by free and informed citizens through a process of general agreement (Scanlon, 1998, p. 153). Such principles would for instance broadly include a right to be alive, to be able to flourish and live a worthy life according to one’s life plan, to be free in one’s movements, to be treated fairly, and so on. For example, when I say that I want to be treated fairly, I imply that I want others to treat me fairly and I can reasonably expect that others want me to treat them fairly as well. In this sense, my right to be treated with fairness is dependent on my duty to also treat others with fairness. One of these mutually agreed upon principles is sometimes referred to as the right to health, or framed slightly differently, the right to be healthy, from which can be derived a right to healthcare (Daniels, 1985, pp. 15–17). Health holds a special place among these fundamental principles, since those who are concerned with one’s ability to carry out one’s own life plans and ideals are ultimately contingent on being in decent health. Health effectively conditions one’s well-being and one’s ability or disability to pursue a life plan, granting it a fundamental position in matters of justice and distribution (Voigt & Wester, 2015). Put differently, being healthy and having access to healthcare safeguard the principle of equality of opportunity (Daniels, 2001).

It is thus reasonable to assume that, under contractualist premises, citizens would agree on the importance of health and healthcare and would thus design their institutions and adapt their behavior accordingly. As has been sketched out above, a right to health necessarily implies a set of duties that aim at guaranteeing the good health of others. This is fairly self-evident, for one can reasonably assess my action of deliberately poisoning the food of my co-workers as wrong and undesirable; it should be rejected according to the mutually agreed principle of being entitled to be in good health. These duties can be positive or negative, they may require one to either do something or abstain from doing some other thing. I may not, for instance, maliciously push a toddler into the pond at the park, but I may, if the risks for my own health are minimal, jump into the pond to save the toddler from drowning. The former duty is negative (ought not to do something) whereas the latter is positive (ought to do something). This latter duty has been described in the literature as the duty of easy rescue (Beauchamp & Childress, 2009, p. 202; Giubilini et al., 2018a, b; Giubilini et al., 2018a, b).

In the context of the current pandemic, individual moral obligations provide an interesting case where the positive duty of easy rescue and the duty not to jeopardize one another’s health end up requiring the same action, as long as these obligations actually actively prevent contagion and thus effectively preserve health. This idea is not new, for it has already been argued before in the context of the Ebola outbreak of 2014–2015 on similar terms regarding quarantine and self-isolation (Giubilini et al., 2018a, b). In the case of the COVID-19 pandemic, it thus appears that the actions of staying at home and limiting one’s movements, promoted by confinement policies, simultaneously fulfill the negative duty of refraining from causing harm that endangers the health of other individuals and the positive duty of easy rescue. It is worth stressing that the harm caused in this situation may be direct or indirect. It is direct when one individual, through careless actions, infects another individual, which may result in a deterioration of their health. It is indirect when, through the same careless actions, that individual infects another individual who ends up hospitalized for treatment, thus draining the healthcare system’s resources, perhaps depriving others in need of care from accessing it. The right to health, in the context of the current pandemic, thus necessarily includes a right not to be infected and, following contractualist theory, a duty not to infect others. Since it can be reasonably assumed that free and informed citizens would reach general agreement on the principle of maintaining good health, and since being infected contravenes the maintaining of good health, it therefore follows that they should also reach general agreement on the principle of non-infection. For the sake of analytical clarity and parsimony, we refer to this principle as the *containment principle*.

### Civil Disobedience

Civil disobedience is a type of protest that takes the form of a communicative breach of the law (Brownlee, 2012a). It consists, at the most basic level, in an individual or a group of people deliberately disobeying the law in order to protest a specific law or order with the aim of bringing change to it. The distinction between civil disobedience and non-civil disobedience (such as radical violence) resides in its *civility*. Following Brownlee,^3^ the civility of an act lies in the motivations of the disobeyer, and more precisely in a “conscientious communicative breach of law motivated by steadfast, sincere, and serious, though possibly mistaken, moral commitment” (2012a, pp. 23–24).

Civil disobedience can be understood as “non-conformity” with what is expected from us as citizens (Brownlee, 2012a, p. 104). In a nearly just society, citizens are submitted to laws that regulate their relation to the State and their fellow citizens. If one has a strong conviction that a law is unjust and that the authorities in charge should revise it, civil disobedience is thus a way of expressing one’s concern. Civil disobedience may be direct (a breach of the law that is denounced) or indirect (the breach of another law) (Rawls, 1999, p. 320). Therefore, when one disobeys the law (no matter which one), she acts as she’s not expected to: she does not conform with the rules. Through her civil disobedience, she informs others about her discontent and also dissociates herself from a law, policy, or event (Brownlee, 2012a, p. 104).

Examples of civil disobedience can be found throughout history and worldwide. Mahatma Gandhi used civil disobedience as an anti-colonialist tool, as exemplified in the Salt March, a pacifist campaign to denounce the salt tax to be paid by the Indian people; in the United States, Rosa Parks refused to give her seat in the bus to a white man as a way to oppose the Jim Crow laws that enforced racial segregation; more recently and in different locations, activists from the movement Extinction Rebellion have frequently blocked roads by gluing themselves to the ground, vehicles, or buildings to call attention to climate change. These cases are commonly understood as acts of civil disobedience, whatever their differences in goals and means.

But how can we determine, one could argue, that these acts are indeed “civil disobedience” and not another form of dissent (conscientious objection, radical violence, mere disobedience)? How can we determine the “civility” of such acts? And then, once the core of civil disobedience is defined, what can be said about its moral justification in society?

#### A Short Overview of the Concept

The birth of the political concept of “civil disobedience” is often attributed to Henry David Thoreau, and more specifically to his essay *Resistance to Civil Government* published in 1849 (later renamed *Civil Disobedience*: Thoreau, 1991). In the early 1840s, this American abolitionist stopped paying the State poll tax as a symbolic gesture to express his disapproval of the State’s support of slavery and the war against Mexico (Alton, 1992; Bedau, 1991, p. 2). Almost a century later, John Rawls was the first to write about the nature and justification of such form of dissent (Bedau, 1991, pp. 10–11). For this reason, Rawls’s definition often constitutes a starting reference. According to Rawls, civil disobedience is “a public, nonviolent, conscientious yet political act contrary to law usually done with the aim of bringing about a change in the law or policies of the government” (Rawls, 1999, p. 320). His conception is thought for nearly just societies, where breaching the law must be an act of last resort (when all the legal means have proven useless) in order to convince policy makers or the responsible institution to consider the claim of the disobeyer. Finally, a last important point is that, according to Rawls, the person who engages in civil disobedience must also be willing to accept the legal consequences that may result from their actions (Rawls, 1999, p. 322). It is useful to stress that whereas the public, nonviolent, and conscientious characteristics of the action pertain to the Rawlsian definition of civil disobedience, the requirements of last resort and of willingness to accept its subsequent legal consequences pertain to its moral justifiability. In contrast to Rawls’s defense of a right to civil disobedience, Joseph Raz defends a right to *political participation*. Raz considers that there is no right to civil disobedience in liberal states on the grounds that such a regime protects a right of political participation by law (Raz, 1979, p. 273). Civil disobedience may only be justified in “illiberal states” for those whose right to political participation is denied. In addition, Raz disagrees with the Rawlsian characteristics of civil disobedience, rejecting both its definition and its conditions for justification, such as the requirement of non-violence, the last resort, or the publicity of the act (Raz, 1979, pp. 267–269). In the same vein as Raz, David Lefkowitz grounds a moral right to civil disobedience as a right belonging to the class of political participation, but he doesn’t restrict it to so-called “illiberal States” (Lefkowitz, 2007, 2018). As a consequence of this moral right to “public disobedience”, Lefkowitz argues that the State should restrain from punishing the disobeyer and favor lighter penalties instead (Lefkowitz, 2007). According to Lefkowitz, if civil disobedience is to be understood as a political right, then the State should not *punish* dissidents, because that would be “equivalent to punishing a person for exercising the right to vote or the right to free speech” (Lefkowitz, 2007). As a result, a penalty such as a fine could seem more appropriate as it would allow the State to publicly penalize the disobeyer while not denying their right to disobey. However, if one takes civil disobedience as a political right seriously (as with the vote or free speech), then one may doubt the reason why the disobeyer should be charged with a penalty for their actions at all (Brownlee, 2018), effectively resulting in a right not to be punished or penalized. Speaking of civil disobedience as a political right thus leads to implausible conclusions. Political rights are after all usually understood as moral rights that should be converted into legal rights or legal rights pertaining to politics, both of which are incompatible with the nature of civil disobedience. In this sense, it is best to understand civil disobedience as a *moral right to political participation*.^4^ Furthermore, if the ultimate goal of the State is to strongly condemn the dissenters, one can easily assume that the authority sees little or no value in the exercise of civil disobedience and does not take seriously the innovative potential of the demands being made. This argument on punishment versus penalization is highly relevant for the normative weight of civil disobedience as a concept for it addresses directly the question of how much should an act of disobedience be punished or penalized relatively to another merely unlawful act.

Brownlee’s extended account of civil disobedience thus allows for the construction of a strong theoretical apparatus, emphasizing civil disobedience as a conscientious and communicative act (Brownlee, 2004, 2012a), more plausible and applicable to real case studies than other conceptions (Brownlee, 2016). For these two reasons, we will be using Brownlee’s approach to civil disobedience in this article. We propose, however, a nuance concerning retributive consequences by arguing that the total absence of punishment or penalization should only occur when an act of civil disobedience is morally justified.

### Brownlee’s Account of Civil Disobedience

Instead of providing us with a strict definition, Brownlee favors a paradigm case approach in which she identifies the main *features* of acts of civil disobedience (Brownlee, 2004). In her view, an act of civil disobedience must include “a deliberate breach of law taken on the basis of steadfast personal commitment in order to communicate our condemnation to a law or policy to a relevantly placed audience” (Brownlee, 2012a, p. 18). Although this may at first sight seem like just another variation of previous accounts, Brownlee’s approach has the advantage of highlighting two fundamental elements of civil disobedience: *conscientiousness* and *communication*^5^ (Brownlee, 2004). Consequently, we will use both features in order to evaluate two cases of breaching the law in times of pandemic. We further define what an act of disobedience requires in order to be qualified as morally justified while remaining consistent with both Brownlee’s theoretical premises and our contractualist background.

#### Conscientiousness

First of all, conscientiousness is to be understood in a descriptive sense (Brownlee, 2004, 2012a, p. 16n2). It is not a virtue or a quality, but rather an attitude that is characterized by its *sincerity* and *seriousness* (Brownlee, 2004). One acts with conscientiousness when one is sincere and serious about their commitment or beliefs (Brownlee, 2004, 2012a, p. 16n2). This should not be confused with *conscience*, which in contrast holds a moral value. Conscience is a “set of practical moral skills” that one needs to cultivate to be “genuinely, self-consciously morally responsive” (Brownlee, 2012a, p. 52). Whereas conscientiousness describes our “conviction” and the strength of our commitment to our own values,^6^ conscience helps us when taking moral decisions by privileging “certain values over others in light of our personal moral situation” (Brownlee, 2012a, p. 10).

Therefore, our conscientiousness and conscience may constitute the trigger for civil disobedience. For instance, if I am convinced that animal experimentation is wrong but the practice is, however, legally permitted, I have a strong incentive for standing up against the law that authorizes animal testing. I know that some laboratories in my university are conducting research through animal testing and that the law as well as an ethical committee allows them to do so. To show my disagreement, I may organize a sit-in in the labs and hold signs with pictures of dead animals and slogans such as “no more killing”, “science without murder”, and so on. It does not mean that my belief or my reflection is right or correct; but it does mean that the actions I undertake are the reflection of my sincerity and seriousness in my commitment to animal rights (Brownlee, 2004). In this example, the “sit-in” is the means I use to share my views with others. In that sense, the fact of communicating my conviction on animal testing is the natural extension of my conscientiousness.

#### Communication

Why is the communicative aspect of civil disobedience so fundamental? For Brownlee, one who overtly states her objection through a breach of the law is actually showing a moral consistency to her commitments (Brownlee, 2004, 2007). In the case of my sincere conviction about animal rights, I am required to state publicly my discrepancy with the law on animal testing with the aim of bringing a change in policy or law, what Brownlee refers to as the “forward-looking element” of communication (Brownlee, 2004, 2007). However, I do not need to have a proposition regarding how the law should be changed, replaced, or repealed. (Brownlee, 2004). My main interest in communicating is first to condemn a law and dissociate myself from it and then to engage my audience (be it the parliament, the university, the scientists, or society as a whole) in a conversation about my disagreement. I want them to recognize that a change in this law is required (Brownlee, 2007). The way in which I communicate is also relevant: what I do (sit-in, interrupt parliament, or block traffic, for instance) and how I do it (publicly or covertly; violently or peacefully; coercively or persuasively). These are what Brownlee calls the “means” and the “modes” of civil disobedience (2004). Although, as said before, authors disagree on the use of violence and the need to make one’s disagreement public,^7^ there seems to be a general agreement that it must be essentially non-coercive^8^ (Brownlee, 2004, 2007). It is worth highlighting that due to the nature of the cases we analyze later on in this paper and the predominant role violence plays in their respective normative assessments, we adopt Brownlee’s conception of civil disobedience since her account is less limiting than Rawls’s regarding the use of violence.

In sum, this rather broad approach puts emphasis on two features that easily combine. The willingness and the effort to engage in a discussion (communication) about our objection reinforces the plausibility of our commitment (conscientiousness).

#### Moral Justification of Civil Disobedience

After reviewing the adequacy of the category of “civil disobedience” we will address a rather normative question about the *justification* for such disobedience, which we already started to tackle in our overview of the concept. The moral justification of civil disobedience is crucial when it comes to punishment. Arguably, civil disobedience should not be punished as a mere illegal act would be. However, the justification of such an act or its absence thereof may also influence its legal, political, or economic consequences. As stated earlier, the way the State punishes, penalizes, or tolerates such actions denotes, in a certain manner, the opinions it holds about the intrinsic value of civil disobedience. In this article, though, we will not seek to discuss the legitimacy of civil disobedience per se, but rather focus on the moral justification of our particular cases. Therefore, we will not ask “can an illegal act be justified?” or “what is the role of civil disobedience in democracy?” but rather: “In the context of a pandemic crisis, is civil disobedience exercised by (a) the healthcare professional or (b) the protester morally justifiable?”.

To evaluate the justification of an act of civil disobedience we will look at its *proportionality*, understood as a relation between the means of protest and their subsequent harms. These concepts are partially taken from Brownlee’s approach and broadly relate to contractualist theory. We argue that what makes an act of civil disobedience morally justifiable is the fact that the means of protest are reasonably appropriate and not excessive and that it implies the minimal amount of harm to others. As we consider that the disobeyers do have legitimate and defensible causes, we suggest evaluating the global proportionality of their claims, understood as a reasonable and appropriate use of means and harms, as a way to establish whether their act is morally justified or not.

The first element, means, is borrowed from Brownlee, for whom they “refer to the types of action that people use to communicate” like words, body movement, facial expression (Brownlee, 2004). “Means” also includes the “strategy” chosen by the disobeyer to communicate (a demonstration, a sit-in, blocking the roads, etc.) (Brownlee, 2007). Secondly, with *harms*, we want to evaluate what harm is, or may be, caused through civil disobedience in our specific cases. Although civil disobedience may not exclude the use of violence, it should nevertheless minimize the potential harm to others. It seems indeed quite reasonable and subject to general agreement to assert that an act is wrong when “it violates basic rights… or expose[s] other people to undue or excessive risks” (Brownlee, 2007). In this sense, part of the justification of civil disobedience also rests on the amount of harm it may generate and the expected good it can provide (Brownlee, 2007). In the end, the evaluation of the relation between means and harms will provide us with the *proportionality* between the act of disobedience, the condemned law, and the goal to be achieved. If this proportionality proves reasonable in those terms, then the act of civil disobedience can be said to be morally justified.

## Two Cases of Civil Disobedience in Times of Pandemic

### An Illustrative Hypothetical Case

Let us first start with a simple hypothetical case to illustrate the mechanisms involved in our theoretical apparatus. Lockdown and confinement policies involve measures that require certain groups of individuals to stay at home and forgo physical attendance at work for the duration of the measures. These measures create a dichotomy among the population, where each worker is then considered as either essential or non*-*essential. Substantially, by essential one should understand that the worker’s professional activity is (1) vital to the minimal functioning of society *and* (2) impossible to undertake without physical attendance. Non*-*essential workers consist of those whose activity does not satisfy these two requirements, and are thus required to stay at home, with the exception of trips required for the fulfillment of vital needs. Essential workers consist for example of healthcare workers, cashiers, delivery drivers, police officers, and so on. It is estimated that if their professional duties are not accomplished, society cannot be functional at a minimal level.

Let us suppose the existence of a worker, Alan, who works as a cashier at his local supermarket. His professional duties, for the sake of simplicity, consist of scanning items’ barcodes, collecting payment from customers, and handing them back change on their payments. His activity is considered essential for it is obviously necessary in order to make sure supermarkets are functional and thus able to satisfy the population’s need for food, drink, and other first necessity products. His activity also necessarily involves close proximity to a large number of customers and occasional physical contact with either them or their belongings should they decide to pay cash. By the nature of his professional activity and its physical requirements, it is fairly straightforward to understand how Alan is both at risk himself and a risk to others in the context of a pandemic of an infectious respiratory disease. Nonetheless, with him being considered an essential worker, there seems to be no other way other than attempting to limit physical proximity as much as possible through protective measures for both himself and customers while maintaining his activity, hopefully lowering the probability of contagion.

Now suppose that the supermarket where Alan is employed is careless about these protective measures. Virtually nothing has changed in his work procedures since the enforcement of confinement policies and no additional measures of protection have been implemented either by the store manager or the company who owns the store. After all, lockdown and confinement policies are implemented by the State and order restrictions on individuals and on which businesses are allowed to open. Protective measures concerning in-house policies are left for essential businesses to decide, as long as they respect the effects of measures that are transversal, for instance a minimum distance between individuals that must be guaranteed. As we have seen, however, this minimum distance cannot reasonably be applied to the clerk–customer relation. Alan is appalled by the carelessness of his bosses and afraid for his own well-being, the well-being of his family whom he lives with, and the well-being of the hundreds of customers he serves every day. He believes that the State should step in and order strict protective measures for supermarkets and their employees, essentially overriding their authority regarding these sorts of internal policies during the crisis. These measures would for instance involve a protective window separating him for the customers, mandatory disinfectant gel for all employees, masks, and so on. He then tries to bring his demands to his managers, only to have them dismissed. He then decides to communicate his claims through an open letter in a local newspaper. He describes what he considers as objectionable working conditions, explains that as long as these are not improved he will strike, and urges the population to support him. Alan therefore decides not to quit his job but to refuse to go to work, as a sign of protest against both his employer’s carelessness and the State’s absence of reaction, which he deems unjust.

Can Alan’s actions be qualified as civil disobedience? To be qualified as such, it must first be made clear that his actions effectively involve a breach of the law, for otherwise he would not be committing disobedience but rather a regular case of protest. It is the case that Alan has breached the law: by refusing to attend work, he has effectively breached the contract between him and his employer. A breach of contract is an unlawful act, and doing so therefore constitutes a breach of the law. As we have highlighted above, a case of civil disobedience must possess two features: it must be made with conscientiousness and it must be effectively communicative. The latter is indeed present; Alan is protesting against both his employer’s and the State’s inaction, and his act of disobedience is at least partly directed towards policymakers who envision security measures during the time of the pandemic. For his action to be qualified as conscientious, it must be shown that it is both consistent with his values and that the means of protest he decided to use must be thought out seriously in terms of how badly it will affect other individuals. As the reason for his protest is the safety of himself and others, the latter requirement is automatically fulfilled, even if his absence implies longer queues at the supermarket. How is his protest consistent with his deeply held values? As we have shown above, the right to health necessarily implies a commitment to the containment principle. Since his reasons for not attending are directly in accordance with what the principle requires, and since the principle should reasonably be endorsed by all persons, Alan is acting consistently with his values. Furthermore, since his protest appeals to values that should reasonably be held by everyone, his reasons may effectively be internalized by policymakers and potentially lead to policy change. In sum, Alan’s protest possesses all the features of a case of civil disobedience and not mere unlawful action. His means of protest can rightly be said to be proportionate as he is in fact minimizing the harm done to others and preserving their basic rights while communicating his political message, therefore morally justifying his civil disobedience.

### Healthcare Professionals

Consider a more sensitive case, that of healthcare professionals. It is self-evident that the medical profession constitutes an essential activity, especially in a time of sanitary crisis. Suppose that a healthcare professional, a doctor or a nurse for instance, proceeds in the same manner as Alan did for substantially similar reasons. It is evident that their concrete demands would differ, for protective measures in a supermarket are not equal to protective measures in a medical setting. Nonetheless, the line of argument put forward by Alan can be in substance put forward by a healthcare professional while using the same means of protest. If a healthcare professional decides not to attend work because of inadequate protection measures, could their action be qualified as an act of civil disobedience?

In order to clear the air, it is first crucial to point out the difference between the concept of civil disobedience and the concept of conscientious objection, since the two are often difficult to distinguish in illegal actions in healthcare (Childress, 1985) and since bioethicists are usually more familiar with the latter. Whereas the conscientious objector seeks to avoid retribution from their breaches of the law on the basis of personal conviction, the civil disobeyer instead not only acknowledges the illegality of their acts but also shows fidelity to the law and is thus ready to face punishment for them (Rawls, 1999, p. 321). Furthermore, the civil disobeyer must hold the particular goal of policy change as a reason for their actions, whereas the objector limits themself to subjective non-compliance (Cooke & Petherbridge, 2016). This transformative feature of the illegal act is, as we mentioned above, crucial to fulfilling the second condition of civil disobedience for otherwise the action possesses no communication at all. The idea behind the healthcare professional’s refusal to go to work at stake here is not simply to avoid risk, but rather to induce a change in policy.

#### Conscientiousness and Communication

There is supposedly no hierarchy among workers that are considered essential. Nevertheless, a healthcare professional’s refusal to attend work seems more problematic than Alan’s refusal to attend his job as supermarket clerk. This is because in addition to the general set of duties that must be performed by all citizens, healthcare professionals, in virtue of their profession, possess an additional *duty of care* towards their patients (also referred to as the *duty to treat*) (Clark, 2005). This deontological duty is surprisingly difficult to ground and it turns out to be difficult to draw its boundaries with precision, especially when confronted with infectious diseases such as the SARS or Ebola epidemics (Brody & Avery, 2009) or, even more stringently, with the current COVID-19 pandemic. The severity of these situations alongside their inevitable reoccurrence has led to a desire to set clear guidelines for healthcare professionals, which are not clearly codified by codes of ethics (Ruderman et al., 2006). What this question is ultimately concerned with is the extent to which healthcare professionals should endanger their own lives for the sake of treating their patients. While some have argued that this extent cannot be properly qualified, either because of the exceptional circumstances where this question arises (Pahlman et al., 2009) or because the current understanding of the consent of healthcare professionals to be confronted with such risks is insufficient (Malm et al., 2008), others have argued for a clear limitation to the duty in these circumstances (Schuklenk, 2020b; Sokol, 2006). The call for a limitation on the duty is grounded in the idea that while it is true that healthcare professionals have certain obligations towards their patients in virtue of their skills and professions, they also possess what has been called *multiple agencies* (Sokol, 2006). The idea of multiple agency states that healthcare professionals are not *only* healthcare professionals, but are also regular citizens with their own rights and duties towards other citizens, including for instance their own families and loved ones (Dwyer & Tsai, 2008). The duty of care is supplementary but not overriding and cannot contradict the basic political and moral rights that belong to healthcare professionals as citizens. They are, after all, part of the general agreement that we discussed earlier. As such, the containment principle applies to them in the same way that it applies to other citizens. It naturally follows that since healthcare professionals possess the same basic rights and duties as other citizens, and since the moral right to political participation is one such right, then they can also exercise their moral right to civil disobedience just like any other citizen. Just like healthcare professionals always retain their right to go on strike (Chima, 2013), the moral right to civil disobedience is a kind of right that cannot be removed from them.

Turning back to the case of the healthcare professional refusing to attend work as a protest against an inadequate working environment due to its lack of protective gear, the intersection between the duty of care, the containment principle, and the political and moral rights of the protester raises a question concerning how conscientious their action can be. Can one consistently abide by the special duty of care that healthcare professionals possess while at the same time exercising a right to disobey through an action that will, by necessary extension, keep some patients from receiving care? As we have shown, the duty of care is in fact not absolute but *pro tanto*. A doctor certainly has a duty of care towards their patients but is certainly not expected to give a kidney to one of their patients in order to improve the patient’s well-being (Sokol, 2006). There is thus no *prima facie* incompatibility between the duty of care and the act of disobeying through the specific means of refusing to go to work. This short demonstration, however, shows merely one thing, namely that it is not necessarily inconsistent to hold the aforementioned beliefs while performing the aforementioned actions, thus guaranteeing that such an act of political protest from healthcare professionals may rightly be deemed to be serious and sincere. Since the protester also has a clear target in mind and since the reasons being appealed to may rightly be understood and endorsed by everyone, their actions qualify as civil disobedience in the descriptive sense. But are they morally justified? Could an argument be made against justification on the ground that while there is no necessary incompatibility between the duty of care and the right of civil disobedience, the harms caused by the means of protest used in this situation are just too great, thus making it disproportionate?

#### Moral Justification

It has been assumed that justifying this means of protest requires a careful balance between the right to civil disobedience and the duty of care, but this need not be the case. Adding the containment principle in the balance lets us see that, in fact, the duty of care may serve as a basis for the present case of civil disobedience. As we have noted, healthcare professionals must abide by the containment principle just like any other citizen. When refusing to attend work, the goal at stake is not merely their right to health and thus not to be infected, as well as that of their loved ones. What is also at stake is their duty not to infect others. As we have shown, in a contractualist perspective these rights and duties cannot be thought of separately. It is thus plausible that the reasons for a healthcare professional not to attend their job go beyond their sole self-interest and include interests for the well-being of their patients, not all of whom are necessarily sick with COVID-19 and thus risk infection from them and their overexposure in inadequate conditions to the disease. In this sense, the means of protest can be justified in virtue of the duty of care itself. Furthermore, a case could be made on the basis that while it is true that a healthcare professional staying at home is not treating any patients, it is also true that a healthcare professional who contracts a fatal case of COVID-19 will never be able to treat any patients ever again. The fear of death is not to be understood solely as a right that healthcare professionals have, even though it indeed provides a strong enough basis for argument in itself. It is also in the public’s interest, especially in times of pandemic, that healthcare professionals be kept as healthy as they can be. A situation where healthcare professionals are decimated by an infectious disease that they were exposed to because of their duty of care is clearly unfair and suboptimal as far as the amount of harm caused is concerned. The protests against the lack of adequate gear aims to prevent just that from happening; they aim at protecting the healthcare professionals’ right to health while at the same time guaranteeing that they may properly accomplish their duty of care towards their patients and not endanger them by their mere presence because of an inadequate clinical environment.

In this way, it can be rightly concluded that refusing to attend work is a morally justifiable means of protesting against inadequate working conditions concerning protection. While doing so certainly causes harms, the harms caused by the absence of this form of protest may reasonably be considered to be possibly greater than in the case of non-protest through such means. The relation between the means and its potential harms shows proportionality and is thus justified. As we have shown, their act of protest can be said to be conscientious for it does not conflict with their deeply held values or basic rights and duties. It also satisfies the requirement of communication for it identifies clear targets and these targets can reasonably endorse the reasons that have led to the act of protest, since they rely on fundamental principles that all individuals ought to adhere to. Finally, it fulfills the criterion of proportionality as we have just argued. Since all three elements are present, their action can effectively be qualified as morally justified civil disobedience.

### Public Demonstrators

Consider now the case of regular citizens who do not have additional duties related to their professional activities. Their job is considered non-essential, and they are thus required to stay at home and are only allowed to go out in order to fulfill vital needs. They are thus asked to self-quarantine on the grounds of the *containment principle.* However, a part of them argues that these measures of confinement are beyond the State’s legitimate use of coercion. They consider these restrictions to be unconstitutional and may in fact constitute an excuse for the State to gain greater control over the population. A demonstration is organized in front of parliament, and about 150 citizens gather together in the square. They carry signs with messages such as “all workers are essential”, “give us liberty”, “I want my life back”, “we all have the right to assemble”, “this is punishment not protection”. They do not respect social distancing, and most of them do not wear masks. Many protesters are live-streaming the demonstration on social media and journalists are covering this event, taking pictures and interviewing demonstrators. As a result, a few citizens are arrested and fined by the police, but the majority simply leaves the square eventually.

#### Conscientiousness and Communication

Do these protesters have a sincere and serious belief that lockdown measures warrant a revision and are the values that support this belief strong enough to defy the lockdown measures in their defense?

It must be assumed that protesters are sincerely committed to the ideas they are pushing forward in their protest. Recall that whether or not their beliefs are erroneous does not matter for the present case, for epistemic correctness is not a feature of conscientiousness, only consistency in commitments is. It indeed seems reasonable to say that these people value their liberty of movement and assembly as well as the possibility of working and earning money as parameters that allow them to live a good life. Through the implementation of the pandemic measures regarding confinement, some of them may have lost their jobs, the self-employed may reasonably be afraid regarding the future of their business, and many of them are possibly physically and mentally suffering by strictly staying at home. In their view, then, the essential parameters of conducting a decent life are constrained, if not totally paused. They understand that the State is taking responsibility for its residents’ health, but they think that this concern simply does not outweigh their personal liberties. They believe that they should make their own decision regarding this exceptional situation. These demonstrators thus balance these exceptional and time-limited pandemic measures with their own values and conclude that they should not conform to the measures. To communicate their disagreement, they directly breach the obligation to stay at home and further disobey its related safety measures, such as social distancing. Some of the protesters are arrested or fined and are therefore penalized for their conduct, which therefore establishes that while confinement measures are not proper law in themselves, they fall in the same analytical category as far as disobedience is concerned since transgressing them is considered unlawful. The choice of location in front of their local parliament building makes it clear that they call on lawmakers and policymakers to revise their decision. The use of signs with short sentences is also part of the performance, as they summarize the claims in a very straightforward manner; the press coverage as well as posts on social media assure the protester that their message reaches different audiences. In that sense, their communicative effort reinforces the plausibility of their commitment. Under this light, it can safely be asserted that protesters against lockdown present both the broad features of conscientiousness and communication, and consequently fit in the category of civil disobedience.

#### Moral Justification

To determine whether or not this act of civil disobedience can be morally justified, we look at its proportionality understood as a relation between the means of protest and its subsequent harms, as we have done in the case of Alan and healthcare professionals.

In the present case, protesters are performing a collective direct breach of the lockdown measures. Although a demonstration does not seem excessive per se, the non-application of safety measures (as a part of the means) may jeopardize the proportionality of the act. Indeed, demonstrators are not only breaching the ban and communicating their judgments, but they are also breaching the containment principle. Note that this lack of proportionality does not imply that the reasons that motivate their actions are illegitimate or invalid, but rather that the means of protest they make use of pose too great a threat to others in relation to those reasons. Through the non-application of safety measures, protesters are not only endangering themselves (which is not problematic as it may be understood as part of their sincerity in commitment), but are also endangering other citizens. This class of other citizens includes all individuals who are not intentionally engaged in the protest and who have not agreed in the first place on this form of risk-taking. They may be harmed directly by being infected by a protester (for instance the police officers who intervene during the protest or passers-by on their way to work), but also indirectly (if a protester is injured and must go to the hospital, it may deprive others in need of care from accessing it). In that sense, disobeying the safety measures shows a discrepancy with the right to health, which we assume is a moral norm that all citizens are subject to and should benefit from, and as such constitutes a disproportionate means.

However, one may argue that demonstrators are indeed disobeying a basic principle, but for the sake of rights that are also considered basic (autonomy, mobility, etc.). According to Brownlee, “an action is wrong to the extent that it violates one moral value or principle but justified to the extent that it respects or fosters another” (Brownlee, 2007). In our case, we are dealing with both the violation of a basic right through inappropriate means, and the intent to foster individual liberties. Our response is that, in the context of a pandemic crisis, violating the containment principle and therefore posing a threat to the basic right to health may jeopardize the future realization of other basic rights (mobility, autonomy, assembly, etc.). Moreover, one must note that these binding measures are limited in time and supported by the containment principle; when the situation goes back to normal, protesters will be granted their individual liberties again. The claims they are currently making on the ground of these liberties call not for their extension (to other people or in terms of content) but for their recovery. This does not mean that such an act of civil disobedience can never be justified. In fact, protesters could have been protesting *within* the frame of safety measures, for instance by gathering in small groups while keeping a physical distance and wearing masks. If performed in this fashion, they would still have been breaching the obligation to stay at home, but without involving other, non-dissenting citizens in the consequences of their acts. On this ground, we conclude that the means of protest used cannot be said to be proportionate for it may result in excessive harms for other citizens. It is true that violence, coercion, or harm have been said to be acceptable to a certain extent or in order for protesters to call attention to their cause. However, still following Brownlee, an act of civil disobedience cannot be justified when it exposes other people to excessive risks or negative consequences (Brownlee, 2007). We therefore conclude that the harms caused are excessive, as they are both direct and indirect, in that they pose a serious threat to non-dissenting citizens’ basic right to health and healthcare. Furthermore, this particular case of civil disobedience is not proportionate to its aims (as liberties will be recovered at some point) or to the law that is being asked to be revised (which is limited in time and favors the preservation of other basic liberties). Therefore, this particular act of civil disobedience cannot be morally justified.

## Conclusion

Throughout this paper we have argued that while healthcare professionals refusing to go to work as a protest against inadequate safety measures can be considered to be an act of morally justified civil disobedience, citizens that participate in public demonstration by deliberately transgressing safety measures in order to protest against confinement measures may also perform an act of civil disobedience, however morally unjustifiable. We have done so by building a theoretical apparatus that draws its inspiration from the philosophical literature on civil disobedience and contractualism, which has helped us formalize the structure of the rights and duties associated with the current pandemic, as far as the preservation of health is concerned. As we have shown, our analysis serves a purpose greater than mere analytical clarification because of the normative features associated with morally justified civil disobedience, mainly that actions that qualify as such may be subject to a lighter retribution should its authors be prosecuted. Our analysis of the rights and duties of healthcare professionals also aims to weaken their image as mere instruments in containing the pandemic. It cannot be stressed enough that doctors, nurses, and all kinds of medical staff are also citizens and persons who, while certainly holding duties towards the general population, also benefit from the same rights that the general population enjoys and may legitimately claim. As crucial as healthcare professionals are in times as trying as the current pandemic, recognizing these rights is the very least that can philosophically, legally, and politically be done as part of, to use T.M. Scanlon’s words, what we owe to them as citizens and individuals.

It must be remembered that the case we have made for civil disobedience rests on the idea of a functioning liberal democracy, which effectively grants legitimacy to the existence of such a right. We have assumed that those who protest against confinement measures do not rely on an informal economy and that staying home is a reasonable action that can be expected of them in order to prevent further harm from happening. The reality is unfortunately much more complex than what we have assumed, which ultimately calls for moral reflection on the feasibility of social distancing and confinement for *all* individuals. As such, our argument may only cover a fraction of the complexity of the issues associated with COVID-19 and its associated governmental responses. Further research and reflection are much needed in order to capture the variety of human experiences in living and complying with State measures and in order to grant these experiences the analytical attention they deserve. We hope that our analysis serves as a step in this much needed direction.

## Footnotes

1 Extracted from the New York Times’s podcast *The Daily*, hosted by Michael Barbaro (20 April 2020) https://www.nytimes.com/2020/04/22/podcasts/the-daily/coronavirus-lockdown-protests.html (Accessed May 6 2020).

2 Stephen Moore is an American television commentator and writer, of libertarian orientation, who publicly argued for the reopening of the economy and against stimulus checks in the context of the COVID-19 pandemic.

3 The content of “civility” is subject to debate, as exemplified by Brownlee’s discussion and critique of Rawls’s definition of civility (which demands non-violence, publicity of the act, and the willingness of the disobeyers to accept the legal consequence of their action) (Brownlee, 2012a, pp. 21–24).

4 We thank an anonymous reviewer for emphasizing the importance of this precision.

5 Brownlee later refined her argument in order to provide the reader with her communicative principle of conscientiousness that links those elements together. This principle is based on four features: consistency, non-evasiveness, universal judgement, and dialogic effort (2012a). Although this reformulation is richer and more detailed, the account she proposes in her 2004 article is sufficient for the exercise we propose here.

6 What Brownlee later refers to as conscientious conviction (2012a, b).

7 For Rawls, civil disobedience must be a non-violent public act and also requires the disobeyer to announce her future breach of the law to the authorities (Rawls, 1999, p. 321). This is in addition to the conditions of moral justification we have highlighted earlier, namely those of last resort and willingness to face the legal consequences of the action. On the contrary, Brownlee states that giving fair notice to authorities may jeopardize the action and thus the effect of civil disobedience. Moreover, she explains that an act can be violent without hurting others and that therefore, to some extent, violence may rightfully be used in some cases (Brownlee, 2004).

8 Although Brownlee recognizes that civil disobedience may have coercing effects irrespective of the disobeyer’s intentions, serious coercive violence is what defines radical protest and not civil disobedience (Brownlee, 2012a, b; see also Brownlee 2007). Moreover, Brownlee argues that coercion is not a convenient strategy for a disobeyer who should rather aim to persuade her audience instead of forcing them to adopt her view (Brownlee, 2007, 2012b, p. 531).
